# CD133 expression predicts lung metastasis and poor prognosis in osteosarcoma patients: A clinical and experimental study

**DOI:** 10.3892/etm.2012.603

**Published:** 2012-06-08

**Authors:** AINA HE, WEIXIANG QI, YUJING HUANG, TAO FENG, JIE CHEN, YUANJUE SUN, ZAN SHEN, YANG YAO

**Affiliations:** 1 Departments of Oncology and; 2 Pathology, The Sixth People’s Hospital, Shanghai Jiao Tong University, Shanghai 200233, P.R. China

**Keywords:** osteosarcoma, CD133, prognosis, lung metastasis

## Abstract

Identifying prognostic factors for osteosarcoma (OS) aids in the selection of patients who require more aggressive management. CD133 has been found to be a prognostic factor of certain tumor types. However, the association between CD133 expression and the prognosis of OS remains unknown. In this study, we analyzed the association of CD133 expression in OS with clinical factors and overall survival, and further investigated its potential role in metastasis *in vitro*. We found CD133 expression in 65.7% (46/70) of OS samples using immunohistochemistry, and it was positively correlated with lung metastasis analyzed by Chi-square test (P=0.002) and shorter overall survival time using the Kaplan-Meier method compared by log-rank test (P=0.000). Multivariate analysis showed that CD133 expression was an independent prognostic factor of patients with OS. To test for direct participation of CD133, we separated CD133^+^ and CD133^−^ cells in the MG63 cell line using magnetic-activated cell sorting and found that CD133^+^ cells were more active in migration by scratch wound-healing assay and invasion by Matrigel invasion assay compared with CD133^−^ cells. Elevated mRNA expression of the stemness gene octamer-binding transcription factor 4 (Oct-4) and NANOG, and the metastasis-related receptor C-X-C chemokine receptor type 4 (CXCR4) were also found in CD133^+^ cells by reverse transcription-polymerase chain reaction. Thus, expression of CD133 was correlated with lung metastasis and poor prognosis in OS patients. CD133^+^ cells may be a type of cancer stem cell with high expression of self-renewal capacity and metastasis-related genes.

## Introduction

Osteosarcoma (OS), as a malignant primary bone tumor, typically occurs in children, adolescents and young adults (1), with an age-standardized incidence of approximately 5 per million per year (2). Despite the improvement of multidisciplinary treatment, including aggressive surgical resection and intensive multiagent chemotherapy and radiotherapy (3), 30% of patients with localized disease and 80% with metastatic disease at diagnosis will relapse (4,5), and 40% of patients succumb to lung metastases (6). Identifying prognostic factors in OS aids selection of those patients for more aggressive management at early time points (7–9).

CD133, also known as prominin-1, is a member of the pentaspan transmembrane glycoproteins (10,11). Although it was initially expressed in hematopoietic stem cells, CD133 presentation was also found in various solid tumors, such as hepatocarcinoma (12), melanoma (13) and synovial sarcoma (14), but not in OS to date. However, Tirino *et al* (15) identified CD133^+^ cells within OS cell lines SAOS2, U2OS and MG-63 in 2008, and other investigators (16,17) have confirmed their presence in various OS cell lines. Furthermore, we investigate the expression of CD133 in OS tissues in this study.

In addition, CD133 has been found to be a prognostic factor for certain cancer types. Several studies have reported that the presence of CD133 in various tumors was correlated with poor prognosis. Song *et al* (18) found that high expression of CD133 was correlated with increased tumor grade, advanced disease stage, elevated serum α-fetoprotein levels and poor survival of patients with hepatocellular carcinoma. Similarly, Horst *et al* (19) found that CD133 expression was an independent prognostic marker for low survival in colorectal cancer. Furthermore, Zhang *et al* (20) reported that CD133 expression was a predictor of poor response to chemotherapy and of reduced disease-free survival time for patients with ovarian cancer. However, Qin *et al* (21) revealed that CD133 was not associated with cisplatin-based chemotherapy resistance or shorter overall survival of patients with advanced serous ovarian cancer. Moreover, Fan *et al* (22) demonstrated that CD133-negative expression correlated with poor prognosis, whereas CD133-positive expression predicted favorable outcome in cholangiocarcinoma patients.

To date, the association between CD133 expression and prognosis of OS remains unknown. In this study, we analyzed the association of CD133 expression in OS with clinical factors and overall survival, and further investigated its potential role in metastasis *in vitro*.

## Materials and methods

### Patient data collection

Paraffin-embedded OS sections from 70 patients who were diagnosed with primary OS and had undergone initial surgery at the Sixth People’s Hospital, Shanghai Jiao Tong University, Shanghai, China, between January 2002 and January 2010 were obtained from the Department of Pathology for immunohistochemical staining. Follow-up information was updated through December 31, 2011, by reviewing medical records and telephone contact. The use of tissue blocks and the chart reviews were approved by the Ethics Committee of the Sixth People’s Hospital, Shanghai JiaoTong University. The relevant clinical data included gender, age, tumor location, tumor size, Ennecking stage, local recurrence status, lung metastasis status and overall survival. Overall survival was calculated as the time from the date of diagnosis to the date of death or the date of last follow-up if the patient was still surviving.

### Immunohistochemistry

Paraffin sections (4-*μ*m thickness) were deparaffinized and treated with 3% hydrogen peroxide for 10 min to quench the endogenous peroxidase activity. Antigenic retrieval was performed by submerging in citric acid (pH 6.0) and microwaving. The slides were then allowed to cool at room temperature, followed by incubation in normal goat serum for 1 h to block nonspecific binding, then incubated overnight at 4°C using CD133 antibody (1:100, Abcam, Hong Kong), and examined using HRP Envision Systems (Dako, Shanghai, China). Rabbit IgG was used as the primary antibody for the negative control. Finally, the sections were visualized after counterstaining with hematoxylin. Each section was evaluated by three independent pathologists without knowledge of the clinical case features. The whole sections were screened for CD133 expression under ×100 magnification.

### Flow cytometry

To measure the proportions of CD133^+^ cells in the human MG63 OS cell line, cells were detached using 0.02% EDTA in phosphate-buffered saline (PBS), counted and washed in PBS. At least 10^5^ cells were incubated with CD133/2(293C3)-APC (1:100; Miltenyi Biotec, Auburn, CA, USA) at 4°C for 30 min in the dark. After washing steps, the labeled cells were analyzed by flow cytometry (Beckman Coulter, Brea, CA, USA).

### Magnetic-activated cell sorting (MACS)

CD133^+^ cells in MG63 were magnetically labeled using a CD133 microbead kit (Miltenyi Biotec) as described in the manufacturer’s instructions. Briefly, 10^8^ cells were dissociated and resuspended in 300 *μ*l PBS supplemented with 0.5% bovine serum albumin (BSA) and 2 mM EDTA (pH 7.2). CD133 microbeads were used for positive selection by MACS cell separation using two MACS MS columns consecutively. After separation by MACS, aliquots of the positive and negative sorted populations were evaluated for purity by flow cytometry. Purities ranged from 90 to 95% for positive and from 89 to 99% for negative populations. During the experiment, 4–6 passages of the sorted cells were used.

### Immunofluorescence staining

CD133^+^ and CD133^−^ cells cultured in 6-well plates were fixed in 4% paraformaldehyde for 30 min at 4°C, washed in PBS, treated with PBS supplemented with 1% BSA for 1 h at room temperature and then stained with CD133 antibody (1:100) at 4°C overnight. Goat anti-rabbit IgG-FITC (CW Biotec, Beijing, China) was used as a secondary antibody, the nuclei were stained with DAPI. Cells were then washed and observed under a fluorescence microscope (Olympus BX41, Tokyo, Japan).

### Western blotting

Protein (50 *μ*g) prepared from MG-63, CD133^+^ and CD133^−^ cells were loaded per lane and electrophoresed in SDS-PAGE, and then transferred onto polyvinylidene difluoride Immobilon-P membranes (Bio-Rad, Hercules, CA, USA) using a transblot apparatus (Bio-Rad). The membranes were blocked in 10 mmol/l Tri-HCl (pH 8.0), 150 mmol/l NaCl and 0.05% Tween-20 (TBST) with 5% (w/v) non-fat milk at room temperature, followed by overnight incubation at 4°C with primary antibodies diluted in TBST [1:1000 for CD133; 1:1000 for β-actin (CW Biotec)]. After washing with TBST, the membranes were incubated for 1 h with an HRP-conjugated secondary antibody diluted 1:5000 in TBST, and the labeled proteins were detected using the enhanced chemiluminescence reagents and exposed to the film.

### Scratch wound-healing assay

Migratory ability was determined using a scratch wound-healing assay. CD133^+^ and CD133^−^ cells were seeded and grown to confluence, and then scratches were made on the cell layer with a pipette tip running across the dishes. Plates were washed twice with fresh medium to remove non-adherent cells. Locations (n=3–4) were visualized and photographed at 0 and 24 h under a phase-contrast inverted microscope (Olympus BX41, Japan). The distance between the two edges of the scratch was measured (23).

### Matrigel invasion assay

Cell invasion was performed using 24-well Transwells (8-mm pore size; Corning, NY, USA) coated with Matrigel (1 mg/ml; BD, NJ, USA) in triplicate. CD133^+^ and CD133^−^ cells (10^5^ per well) were seeded in the upper chambers in culture media containing 0.2% fetal bovine serum (FBS), and the lower chambers were filled with 500 *μ*l 10% FBS medium to induce cell migration. Following incubation for 24 h, cells inside the chamber were wiped off with a cotton swab, invading cells were stained with Giemsa (Lexiang Biotec, Shanghai, China) and examined using microscopy (Olympus BX41). Cells in at least six random microscopic fields (×200 magnification) were counted to determine relative invasive potential.

### Reverse transcription-polymerase chain reaction (RT-PCR)

Total cellular RNA of CD133^+^ and CD133^−^ cells was extracted using TRIzol reagent (Ambion, Austin, TX, USA) and treated with RNase-free DNase (DNase I, Ambion) to remove potential genomic DNA contaminants. Total RNA (1 *μ*g) was reverse-transcribed with the RETROscript^™^ Two-Step RT-PCR system (Ambion). Reactions were performed according to the manufacturer’s instructions using SYBR green PCR supermix (Sangon Biotec, Shanghai, China) in a single-color RT-PCR detection system (Stratagene, Santa Clara, CA, USA). The gene expression levels (mRNA) of octamer-binding transcription factor 4 (Oct-4), NANOG, and metastasis-related receptor C-X-C chemokine receptor type 4 (CXCR4) were normalized to that of the GAPDH transcript. Sequences for mRNAs from the nucleotide data bank (National Center for Biotechnology Information, USA) were used to design primer pairs for RT-PCR reactions (Table I).

### Statistics

Correlations between CD133 and clinicopathological features were examined by the Chi-square test. Survival rate was calculated using the Kaplan-Meier method. Univariate and multivariate survival analyses were performed to test the association of clinicopathological features with OS, incorporating log-rank testing and Cox proportional hazard regression models. Comparison of the two experimental groups was performed using the independent-samples T-test. Statistical analyses were conducted using SPSS 16.0. Data were expressed as the mean ± SEM. A value of P<0.05 was considered to indicate statistical significance.

## Results

### Patient clinical characteristics

As shown in Table II, there were equal numbers of male and female patients, and 29 (41.4%) patients were older than 18 years of age. Tumors were located in axial (1/70), upper limb (5/70) and lower limb (64/70) locations. In total, 20 (28.6%) and 54 (77.1%) patients had local recurrence and lung metastases, respectively. Regarding Ennecking staging, 58 patients were at stage II and 12 at stage III. Over the course of the study, 63 patients succumbed to tumor-related causes. The median overall survival of patients was 20.0 months [95% confidence interval (CI), 16.6–23.4 months].

### Correlation between CD133 expression and clinicopathological characteristics

**Figure 1:**
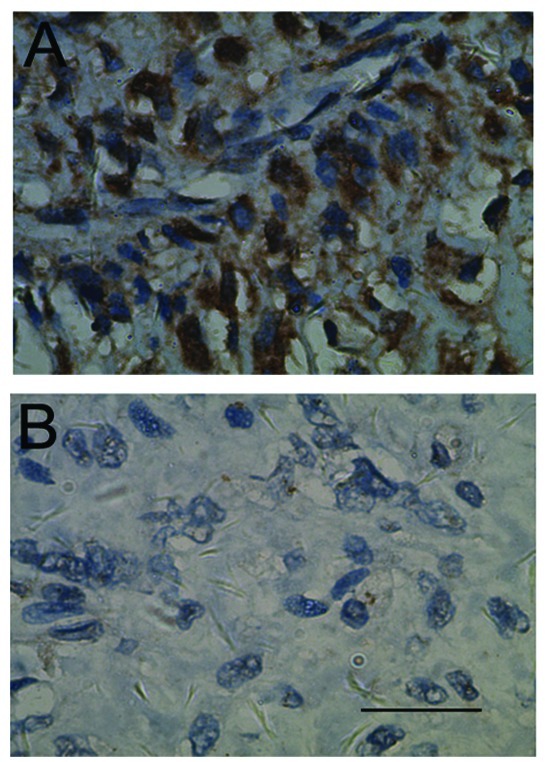
Immunohistochemical analysis of CD133 staining. (A) Positive; (B) negative. Scale bar, 100 *μ*m.

CD133 stained the cytoplasm and membrane of tumor cells and representative images of immunostaining of OS tissues are shown in Fig. 1. Cases were defined as CD133-positive if CD133 staining was detected in >10% of the entire tumor area (21,24).

As shown in Table II, CD133 expression was found in 46/70 (65.7%) of OS samples. Of the 54 patients who developed lung metastasis, 40 cases were in the CD133-positive group, whereas only 14 cases were in the negative group (P=0.002), indicating that CD133 expression was positively correlated with lung metastasis as analyzed by the Chi-square test. However, no significant association was observed between CD133 expression and any of the other clinicopathological characteristics listed in Table II.

### Correlation between CD133 expression and prognosis of OS patients

**Figure 2:**
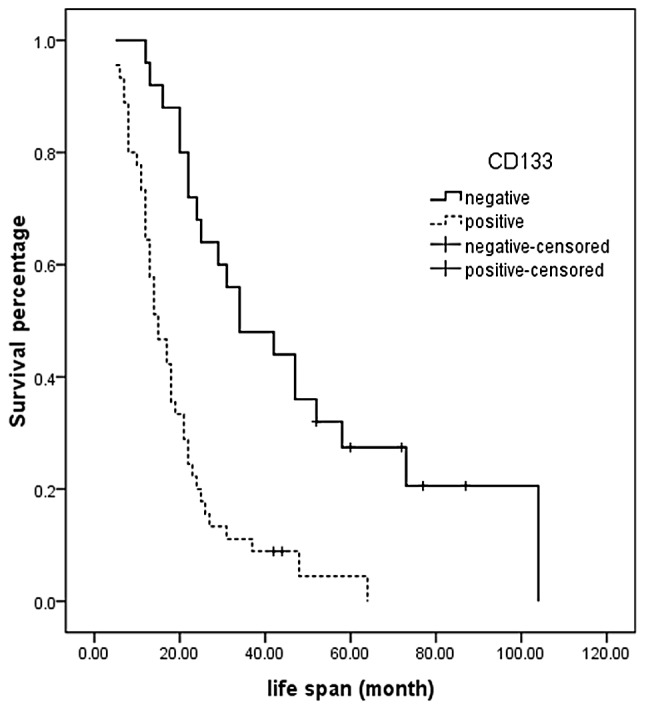
Log-rank survival analysis of osteosarcoma patients according to CD133 expression.

At the cut-off date, the median overall survival was significantly shorter in the CD133-positive group compared with the CD133-negative group (34.0 months; 95% CI, 16.0–51.9 vs. 15.0 months; 95% CI, 11.3–18.7; P=0.000) (Fig. 2). Univariate survival analysis showed that the significant prognostic factors were tumor size, local recurrence, lung metastasis and expression of CD133. Multivariate analysis showed that the CD133 expression and tumor size were independent prognostic factors of patients with OS (Table III).

### Identification of CD133^+^ and CD133^−^ cells

**Figure 3:**
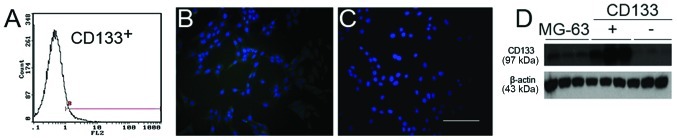
Identification of CD133^+^ and CD133^−^ cells. (A) Detection of CD133^+^ cells in the MG-63 cells by flow cytometry. (B) CD133^+^ cells under fluorescent light. (C) CD133^−^ cells under fluorescent light. Scale bar, 100 *μ*m. (D) Expression of CD133 protein in MG-63, CD133^+^ and CD133^−^ cells, as analyzed by western blotting. β-actin was used as an internal control.

The patient data showed that expression of CD133 was related with lung metastasis in OS. To investigate the potential mechanisms, we sorted the OS cell line MG-63 for CD133^+^ and CD133^−^ populations using MACS, and further confirmed our results using immunofluorescence staining and western blotting (Fig. 3).

### Migratory ability of CD133^+^ and CD133^−^ cells

**Figure 4:**
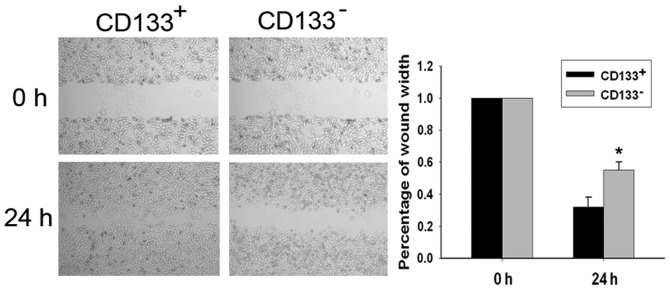
Migratory ability of CD133^+^ and CD133^−^ cells. The distance between the edges of the scratch became increasingly narrow in the CD133^+^ cells. There were notable differences between the groups at 24 h. Data are shown as the mean ± SEM from three separate experiments. ^*^P<0.05.

Migratory ability of the CD133^+^ and CD133^−^ cell populations was examined using a wound-healing assay (Fig. 4). Following incubation of physically wounded cells for 24 h, CD133^+^ cells were found to have traveled a significantly longer distance than CD133^−^ cells (P<0.05). The percentages of wound width were 32.00±6.11 and 54.67±5.21%, respectively.

### Invasiveness of CD133^+^ and CD133^−^ cells

**Figure 5:**
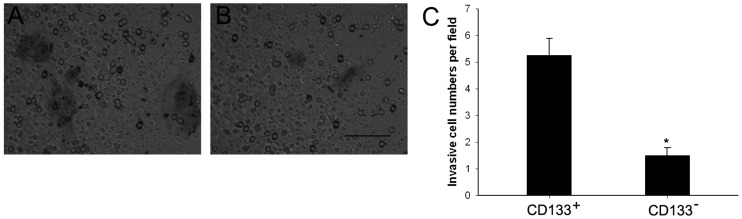
Invasive ability of the CD133^+^ and CD133^−^ cells. The membranes were stained with Giemsa after removal of cells from the chambers [(A) CD133^+^ cells; (B) CD133^−^ cells)]. Scale bar, 100 *μ*m. (C) Quantitative measurement of the invaded cells. Data shown as mean ± SEM from four separate experiments. ^*^P<0.05.

To analyze invasiveness of the CD133^+^ and CD133^−^ cell populations, we performed Transwell invasion assays using cell culture inserts covered with extracellular matrix components. Cells (5.25±0.63 per field) traveled through the membranes in the CD133^+^ group, compared to only 1.50±0.29 cells per field in the CD133^−^ group (P<0.05) (Fig. 5).

### mRNA expression of Oct-4, NANOG and CXCR4

**Figure 6:**
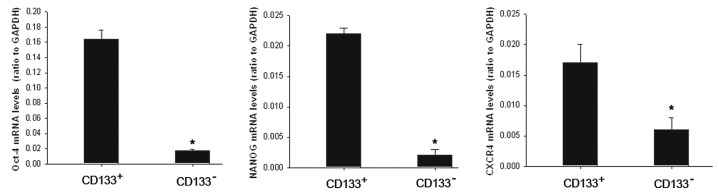
RT-PCR analysis of Oct-4, NANOG and CXCR4 in CD133^+^ and CD133^−^ cells. Data are shown as the mean ± SEM from three independent experiments. ^*^P<0.05.

As CD133 has been considered to be a cancer stem cell marker in several tumor types, we examined mRNA expression of the stemness gene Oct-4, NANOG and metastasis-associated receptor CXCR4 using RT-PCR in the CD133^+^ population and its counterpart CD133^−^ cells. The results showed that mRNA expression was significantly higher in the CD133^+^ population (Fig. 6).

## Discussion

OS is a highly aggressive tumor, comprising approximately 20% of all bone tumors and 5% of pediatric tumors (25). However, our current understanding of OS etiology is limited. Previous cancer studies have shown that many tumors contain a small population of cells termed cancer stem cells (CSCs), which are responsible for tumor progression, metastasis, recurrence and resistance to chemotherapy and radiation treatments (26,27). While the experimental evidence for the existence of CSCs was first proposed for hematological malignancies, more recently CSCs have been observed in solid tumors, including breast, brain, pancreatic and bone cancers (28). The existence of CSCs in primary OS and cell lines derived from human OS was previously demonstrated (15,29).

CD133 has been considered as a CSCs marker in a number of tumor types (28,30,31), such as colorectal, brain, prostate, pancreatic and gastric cancers, and also in OS. Subsequent studies have confirmed that CD133^+^ cells from OS cell lines showed stem-like features including high proliferation rate, cells detected in the G2/M phase of the cell cycle and Ki-67 positivity (15). In our patient data, we found that CD133 was a worse prognostic factor for OS and lung metastasis. *In vitro*, we found that CD133^+^ cells efficiently invaded and migrated, according to high expression of Oct-4, NANOG and CXCR4. These findings support the proposed link between CD133 and CSCs.

Oct-4, also known as Oct-3, Oct-3/4 and POU5F1, is one of the earliest transcription factors expressed in the embryo, and has been identified as fundamental to the maintenance of pluripotency and self-renewal in embryonic stem cells and primordial germ cells (32,33). NANOG is also a homeodomain transcription factor thought to be a key factor in sustaining the pluripotency of embryonic stem cells (34). A recent study of Oct-4 and NANOG in OS demonstrated that the two markers were highly expressed in CSCs, which suggests that these transcription factors play a role in sarcoma stem cell biology (35). CXCR4, a chemokine receptor in the GPCR gene family, has been proven to play an essential role in the metastasis of CSCs (36). OS stem cells were found to express more CXCR4 than normal tumor cells (37).

In conclusion, our findings revealed for the first time that high expression of CD133 in OS tissues indicates a high lung metastasis risk and short survival time in OS patients. CD133^+^ cells were more active in invasion and migration than CD133^−^ cells, in accordance with higher expression of Oct-4, NANOG and CXCR4. These findings support the proposed link between CD133 and CSCs. However, further animal experiments using *in vivo* cell xenografts are warranted to confirm this. Prognostic judgment in order to improve the effect of treatment on OS and more suitable treatment strategies including the application of CD133 target gene therapy could be accomplished according to the expression status of CD133.

## Figures and Tables

**Table I:** List of primer sets used in this study.

Gene (GenBank accession no.)	Sequence (5′ to 3′)	Tm (°C)	Location
Oct 4 (NM_002701)			
Forward	CTTGAATCCCGAATGGAAAGGG	61	42–63
Reverse	GTGTATATCCCAGGGTGATCCTC		205–183
NANOG (NM_024865)			
Forward	TTTGTGGGCCTGAAGAAAACT	61	83–103
Reverse	AGGGCTGTCCTGAATAAGCAG		198–178
CXCR4 (NM_003467)			
Forward	TGACGGACAAGTACAGGCTG	61	215–234
Reverse	AGGGAAGCGTGATGACAAAGA		277–257
GAPDH (NM_002046)			
Forward	AAGGTGAAGGTCGGAGTCAAC	61	7–27
Reverse	GGGGTCATTGATGGCAACAATA		108–87

**Table II:** Correlation between CD133 expression and clinico-pathological factors in the osteosarcoma patients.

Variable	n	CD133		P-value
		Positive	Negative	
Gender				
Male	35	26	9	0.131
Female	35	20	15	
Age				
≤18 years	41	24	17	0.233
>18 years	29	21	8	
Tumor location				
Axial	1	1	0	0.773
Upper limb	5	4	1	
Lower limb	64	40	24	
Tumor size				
<10 cm	38	22	16	0.224
≥10 cm	32	23	9	
Ennecking stage				
II	58	35	23	0.130
III	12	10	2	
Local recurrence				
Yes	20	13	7	0.937
No	50	32	18	
Lung metastasis				
Yes	54	40	14	0.002
No	16	5	11	

**Table III:** Multivariate Cox regression analysis of potential prognostic factors for osteosarcoma patients.

	B	SE	Wald	df	Sig.	Exp (B)	95% CI for Exp (B)	
							Lower	Upper
CD133 expression	1.135	0.310	13.391	1	0.000	3.112	1.694	5.716
Local recurrence	0.438	0.287	2.320	1	0.128	1.549	0.882	2.720
Lung metastasis	0.336	0.338	0.984	1	0.321	1.399	0.721	2.716
Tumor size	0.551	0.266	4.287	1	0.038	1.734	1.030	2.921
